# Household Hints and Recipes

**Published:** 1878-10

**Authors:** 


### Household Hints & Recipes.

ORANGE WHEY.—Boil a pint of fresh milk; when sufficiently boiled add to it the expressed juice of an orange, with a small part of the peel, let stand until it curdles. Strain for use.

---

REMEDY FOR RATS.—Peppermint is said to be so obnoxious to rats that they will not long remain where it is plentifully used. A farmer drove a horde of rats from his barn by binding up mint with his wheat.

---

SHAMPOO-FLUID.—Boil ¼ lb. soap-wax in 6 to 8 pints of water; when cool, add 2 oz. sal-ammoniac, and 3 oz. best potash. Dilute with 1½ to 2 pints of water, and perfume with a few drops of some essential oil on a lump of sugar.

---

RAISED WAFFLES.—Melt five ounces of butter in one pint new milk, when cool beat in two and a half teacupfuls of sifted flour, and not quite one gill yeast. Allow six hours for rising. Just before baking beat the yolks of four eggs and stir in thoroughly to the batter, then the whites, beaten stiff and stirred in last.

---

IN CANNING FRUIT.—Either put glass jars into a pan of cold water and bring the water to scalding heat with the jars in it, emptying each as it is wanted, or wrap a dish towel wrung out of cold water round the jars while filling, and you need not fear breaking them by putting boiling fruit in them.

---

FLY-GLUE.—
Rosin, 4 oz.
Raw linseed oil, 1 oz.
Melt; add,
Honey, ½ oz.

Stir well and spread on paper, which previously has been drawn through a solution of alum, else the "glue" goes through.

---

WATERPROOF PAPER.—Sheets of stout manila passed through a hot bath of aqueous solution of zinc chloride (at 75° B.), pressed strongly together and then soaked in dilute aqueous soda solution containing a small amount of glycerine, cohere to form a strong, stiff, water-proof board admirably adapted to the construction of small boats. Single sheet of paper passed quickly through the zinc chloride bath, pressed and washed and dried, are waterproof, and may be otherwise joined to form waterproof boards by any suitable cement.—*Scientific American.*

---

TO REMOVE INK.—The following methods we have not tried, but they are said to be infallible: "To extract ink from cotton, silk, and woolen goods, saturate the spots with spirits of turpentine, and let it remain several hours; then rub it between the hands. It will crumble away without injuring the color or the texture of the article. To extract ink from linen, dip the stained part in hot tallow; when cool, wash the garment in soapsuds, and the ink will disappear."

---

HOW TO GET RID OF STUMPS.—General Colquitt, of Georgia, in a recent address, said: "To remove stumps from a field, all that is necessary is to have two or more sheet-iron chimneys, some four or five feet high. Set fire to the stump and place the chimney over it, so as to give the requisite draft at the bottom. It will draw like a stove. The stump will soon be consumed. With several such chimneys of different sizes, the removal of stumps may be accomplished at nearly nominal labor and expense."

---

A NEW TANNING PROCESS.—M. Charles Paesi, an Italian chemist, has discovered a new mode of tanning, which is stated by the *Journal d'Hygiene* to be much superior in its results, as well as more expeditions than any mode in which tan bark is used. It consists in macerating the skins in a bath of perchloride of iron and sea salt dissolved in water. The operation lasts from four to six months. The perchloride is a powerful disinfectant, and is said to render the industry much more healthy than it now is.

---

PAINTING IRONWORK.—The best paint for iron work is either the oxide of iron paint, known as the Torbay paint, or the silicate oxide paint, both consisting of oxide of iron and silicious matter, to which any color may be added and applied in the usual way. They can be applied even after the surface has commenced to rust, as from their nature they amalgamate freely with the rust, forming an impervious coating adhering well to the surface, and yet sufficiently elastic to prevent cracking when the iron expands or contracts under variations of temperature. Bituminous or tar mixtures, thinned with linseed oil, are well adapted for iron-work, especially when they can be applied hot, or to the heated surface of the metal, so as to insure a firm adhesion by entering the pores. A mixture of cilicate oxide with tar also forms a good durable coating on iron. When ironwork is to be painted with ordinary lead paint red lead should be used. The adhesion of such a coating on iron work can seldom be depended on in consequence of the non-porous surface. This is further prevented by the galvanic action that sets in between the iron and the lead. Galvanizing, or coating the surface with a preparation of zinc, is also frequently resorted to as a preservative. With all such coatings the surface must be perfectly clean and free from rust. It is advisible, so as to prevent rusting, that all ironwork should be coated with some preservative soon after it leaves the mould, forge, or mill.

---

INVISIBLE INK FOR POSTAL CARDS.—The *Deutsche Illustrirte Gewerbezitung* proposes the use of what may be called "postal card ink," for messages which are sent on such cards or otherwise unsealed. A solution of nitrate or chloride of cobalt, or chloride of copper, mixed with a little gum or sugar, produces a "magic ink," which is made visible by warming, either by holding against the stove or over a burning match. Potassium ferrocyanide in solution may also be used; but this requires a developer, for which either copper or iron sulphate may be employed. With the former the writing will appear in brown, and with the latter in blue color.

---

DRYING OIL.—Linseed Oil may be rendered drying, without boiling, by the following process: Mix linseed oil, the older the better, with two per cent of borate of manganese, and heat the mixture on a water or steam bath to 100° or at most to 110°. The product is an excellent, light-colored, rapidly drying oil. By frequently stirring the mixture, the drying property of the oil is increased. The borate of manganese is easly procurable in commerce, but it can be readily prepared by precipitating a solution of sulphate of manganese by one of borax, washing the precipitate and drying it at the ordinary temperature of the air, or with a gentle heat, 100° or thereabouts.

---

TO IMITATE GROUND GLASS.—Put a piece of putty in muslin, twist the fabric tight, and tie it into shape of pad; well clean the glass first, and then apply the putty by dabbing it equally all over the glass. The putty will exude sufficiently through the muslin to render it apaque. Let it dry hard and then varnish. If a pattern is required, cut it out on paper as a stencil plate, and fix it on the glass before applying the putty, then proceed as above; remove the stencil when finished. If there should be any objection to the existence of the clear spaces, cover with slightly opaque varnish.

---

TO FILTER LARGE QUANTITIES OF WATER.—The following is recommended as giving satisfaction. Take a tub, or the half of a barrel cut in two, and bore in the bottom a number of holes of about a quarter of an inch in diameter; over them lay a piece of Canton flannel with the downy side up. Cover this with a layer of three or four inches of clean white sand, followed with about two inches of coarsely ground charcoal, and lastly four inches of the same white sand. The water to be filtered must be poured on gently so as not to disturb the different layers. It is sometimes necessary to strain again the first portions, but after a short time, unless the supply is unusually foul, large quantities of clear water are rapidly obtained. The charcoal and the cloth have to be occasionally renewed.

---

HOT WATER AS A RESTORATIVE FOR PLANTS.—M. Willermoz, in the French "Journal of the Society of Practical Horiculture," relates that plants in pots may be treated with hot water when out of health, the usual remedy for which has been repotting. He says that when ill health ensues from acid substances contained or generated in the soil, and this is absobred by the roots, it acts as a poison. The small roots are withered and cease their action, consequently the upper and younger shoots of the plant turn yellow, and the spots with which the leaves are covered indicate their morbid state. In such cases the usual remedy is to transplant into fresh soil, clean the pots carefully; secure good drainage, and often with the best results. But the experience of several years has proved with him the unfailing efficacy of the simpler treatment, which consists of watering abundantly with hot water at a temperature of about 145° Fahrenheit, having previously stirred the soil of the pots so far as might be done without injury to the roots. Water is then given until it runs freely from the pots. In his experiments the water first came out clear, afterwards it was sensibly tinged with brown, and gave an appreciable acid reaction. After this thorough washing the pots were kept warm. Next day the leaves of two Ficus elastica so treated ceased to droop, the spread of black spots on the leaves was arrested, and three days afterwards, instead of dying, the plants had recovered their normal look of health. Very soon they made new roots, immediately followed by vigorous growth.—*Western Review.*

